# Supplementary material for: Moment-by-moment tracking of naturalistic learning and its underlying hippocampo-cortical interactions
Source: Nat Commun. 2021 Sep 13;12:5394. doi: 10.1038/s41467-021-25376-y (PMC8438040; doi:10.1038/s41467-021-25376-y)
Supplement: Supplementary file 1 — Supplementary Information [file 41467_2021_25376_MOESM1_ESM.pdf]

Supplementary Information | Moment-by-moment tracking of naturalistic learning and its underlying hippocampo-cortical interactions

1. MichelmannSI.pdf

Supplementary Methods, Notes, Figures, Legends and Tables

Supplementary Methods, Notes, Figures, Legends and Tables provide additional analyses, visual illustrations of statistics and electrode placement and information on patient details.

Supplementary Information | Moment-by-moment tracking of naturalistic learning and its underlying hippocampo-cortical interactions

Supplementary Methods

Supplementary Method 1: Distribution of consensus on event boundaries between raters In order to compare consensus between the first and second run of boundary norming, the distribution of agreement was compared between the 2 runs (agreement at each moment is the proportion of participants that marked that moment as an event boundary). In a first step, the quantiles of the distributions (across time) were plotted against each other in a Q-Q plot. We then analyzed the distribution of the data by binning the moments in the story into 232 bins, derived from the Freedman-Diaconis rule: $2\frac{\mathrm{IQR}(x)}{\sqrt[3]{n}}$. The difference in distribution was thereafter compared by assessing their kurtosis. Statistics on the kurtosis were computed on the difference between the first and the second run. The true difference was compared to the distribution of differences of 1000 randomly assigned labels; a p-value was derived as the ratio of differences that were bigger under such random permutation (Supplementary Fig. 1b, right).

Supplementary Method 2: Similarity in meaning of word predictions in the replication of the word-prediction experiment In the main text, the behavioral analysis of prediction accuracy used a binary dependent measure (i.e., was the correct word predicted or not). Here, we explore a more continuous measure of accuracy, where we compute the cosine similarity between the Glove vector embeddings of the correct word and the predicted word. Specifically, we used the 300-dimensional 42B vectors that were pre-trained on common crawl[81]. These parametric estimates are arguably more sensitive than the binary measure used in the main text, insofar as they give partial credit if the predicted word is semantically similar to, but not the same as, the correct word (e.g., if the participant predicts 'journalist' instead of the correct word 'reporter'). Statistically, the average cosine similarity between word embeddings of predicted and correct words was compared between the two groups (i.e., participants who had and had not heard the story before) with a dependent sample t-test. Additionally, each subject's average cosine similarity between word embeddings of their predictions and the correct words was compared between the two groups with an independent sample t-test.

Supplementary Method 3: High frequency power around word onsets To determine the peak response in high-frequency activity ($70-200Hz$) to word onset, we applied a multitapering approach with a window width of $200ms$ and three orthogonal Slepian tapers, i.e., a spectral smoothing of $10Hz$. Power was estimated in steps of $5Hz$, omitting the $120Hz$ and $180Hz$ band (as per harmonics of line noise). Averaged power was subsequently locked to the onset of words, data were z-scored across time and averaged across all words and channels that displayed a prediction effect.

Supplementary Method 4: Additional correlations between behavior and neural predictive recall As an additional way of relating event boundaries to neural predictive recall, we computed the cross-correlation of these two measures. The time-course of agreement (of button-presses indicating event boundaries) was down-sampled to the neural time-course of prediction and correlated. The neural time-course of prediction was shifted against the behavioral time-course to identify the lag that yielded the peak correlation. In a separate analysis, we also examined the correlation between neural predictive recall and the change in behavioral next-word prediction accuracy, this time using the cosine-similarity behavioral prediction measure described above (instead of the binary measure used in Fig. 4c in the main text). Cross-correlations were compared statistically to 1000 cross-correlations with phase-shuffled data; p-values were derived as the proportion of random correlations that were higher than the true correlation; p-values were corrected for multiple comparisons by controlling the false discovery rate[59].

Supplementary Notes

Supplementary Note 1: Further evidence for increased consensus on event boundaries upon one-shot learning

We predicted that subjects would have a better understanding of the underlying event structure of the story on the second run, and therefore would agree more about event boundaries on the second compared to first run, despite not knowing of each other's responses and not receiving feedback about the accuracy of their responses. This change in the distribution of agreement is visible in a quantile-quantile plot (Supplementary Fig. 1a): The deviation from a diagonal line reflects a relative over-representation of moments of high agreement on the second run. Statistically, more accurate responses on the second run also result in more observations in the tails of the agreement distribution. In keeping with this, kurtosis on the second run (24.482) was higher than the kurtosis on the first run (17.94, Supplementary Fig. 1b, left) and this difference (6.512) was significantly higher than expected by chance $p = 0.036$, i.e., under random assignment of run-labels (1000 permutations, Supplementary Fig. 1b, right).

Supplementary Note 2: Unique characteristics of consensus learning

In the measure of consensus learning, we find that the mean difference in cosine similarity between the run 1 and run 2 corresponds to 0.209 standard deviations across raters, which is a 'small' effect according to Cohen's d. Furthermore only 126/205 participants increased in consensus to others. There are, however, some noteworthy characteristics of consensus learning that should be considered: A decrease in consensus does not necessarily mean that a participant did not learn anything: If different participants speed up their event boundary detection (from run 1 to run 2) to different degrees, due to different amounts of learning, this will work against finding an increase in consensus (some participants may find it hard to 'keep up' with the rest of the sample). Indeed, an additional 22 participants that did not increase in similarity to others on the second run nonetheless increased in similarity to others from the first run. When compared to the 57 remaining participants that decreased in similarity to both runs, these 22 participants were also more similar to others on the second run ($t(77) = 3.625$, $p < 0.001$). This leads us to believe that at least some of these additional participants improved their responses, but could just not keep up with the overall trend. The principle that the overall learning rate will 'raise the bar' for all participants also holds for the measure of average cosine similarity (where it also leads to an underestimation of the true learning rate and effect size); it is something that is unique to the measure of consensus as a proxy for learning.

Supplementary Note 3: Further evidence for one-shot learning of word predictions

In the replication experiment of behavioral prediction learning, we could analyze the quality of individual word predictions by computing the similarity between predicted word and correct word via the cosine-similarity between their 300 dimensional Glove vector embeddings (pre-trained on common crawl)[81]. This average similarity was higher in the group that had listened to the story before ($t(959) = 34.967$, $p < 0.001$, $d = 1.127$, Supplementary Fig. 1d, purple line), providing converging evidence that participants were learning to predict the next word based on prior exposure to the story. In the replication experiment we could also compare subjects' prediction performance between groups. Subjects' vector embeddings of their predictions were more similar to the correct words ($t(98) = 6.512$, $p < 0.001$, $d = 1.316$) if they had heard the story before.

Supplementary Note 4: Cross-correlation between neural predictive recall and event boundary time-course

To further test whether predictive recall encompasses the structure of the story, thereby allowing for the anticipation of boundaries, we assessed cross-correlation between the behavioral time-course of agreement (Fig. 1a) and the neural time-course of memory-based prediction (Fig. 4a). When we correlated the average time-course of agreement from the first and second behavioral run with the neural time-course of memory-based prediction, we obtained a maximum correlation of $r = 0.217$ at -1.69 seconds (i.e., before boundary detection); note that this negative lag is expected because a behavioral response can only happen after an event has been neurally registered. This correlation was significantly higher than what we obtained with phase-shuffled time-courses of memory-based prediction; several lags survived a false discovery rate multiple comparison correction ($p_{FDR} = 0.002$, 1000 permutations, Supplementary Fig. 4a). If behavioral learning leads to a more accurate representation of an underlying event structure, we further expected that the time-course of agreement derived from the second behavioral run should correlate slightly better with the neural data. Indeed, with the second behavioral run ($r = 0.221$, peak: $-1.61s$) the maximum correlation was slightly higher than the with the first behavioral run ($r = 0.197$, peak: $-1.76s$), however, only a statistical trend was observed when this difference (0.024) was compared to differences of randomly re-sampled behavioral groups ($p = 0.063$).

Supplementary Note 5: Further analyses linking neural predictive recall to one-shot learning of story content

In the main text, we report that the maximum correlation between neural predictive recall and (behavioral) predictive recall of individual words in the "replication" experiment ($r = 0.113$) was found at 590ms after word onset ($p_{FDR} = 0.006$, Fig. 4c, fuchsia). When we repeated this analysis using cosine-similarity to measure predictive recall of individual words (instead of the binary measure used in the main text), the maximum correlation ($r = 0.127$) was found at 320ms after word onset $p_{FDR} = 0.007$ (Supplementary

Fig. 4b, purple). This more sensitive analysis therefore converges with the lag found in the original behavioral prediction experiment (Supplementary Fig. 4b, turquoise).

Supplementary Note 6: Gamma response to word onset on CPR-channels

The neural high frequency response (70 – 200Hz) to word-onset peaked at 320ms (run 1) and at 389ms (run2, Supplementary Fig. 4b). These lags correspond closely to the lag obtained in the preceding analysis (i.e., when we looked for the maximal cross-correlation between the time courses of neural and behavioral predictive recall), suggesting that neural learning entailed predictions about individual words via anticipation of the neural high frequency response to word-onset.

Supplementary Note 7: Feature analyses of Multivariate Mutual Information related to predictive recall

In order to clarify which frequency bands were most relevant for the increased multivariate mutual information at moments of predictive recall (compare Fig. 5a, right), we repeated this analysis omitting each feature in the hippocampus (Supplementary Fig. 5a). This analysis demonstrates how much the overall effect diminishes if each feature is removed from the analysis. We further recomputed the MI for each hippocampal feature in isolation (Supplementary Fig. 5b). Mostly the raw signal and low frequencies ($< 35Hz$) shared information with cortex.

Supplementary Note 8: Characterization of event boundaries in the story

In order to demonstrate that participants' responses capture meaningful moments in the story, we characterize the properties of our event boundaries. We further plot the 4680 individual responses from the second behavioral run that were used to define event boundaries, as a raster-plot together with changes in the story (Supplementary Fig. 6a).

Story content Based on the event indexing model[82] an independent rater (C.K.S.) marked each clause of the story and decided whether there was a change in time, space, object, character, cause or goal. She further assigned the marked event boundaries to those clauses. We found that the presence of an event boundary within a given clause is associated with upcoming changes in the next clause, which suggests that participants were able to perform the task and mark "moments where one natural and meaningful unit ends and another begins" : Across 146 clauses we assessed the phi coefficient for a binary association between the presence of an event boundary and a change in each of the respective dimensions across clauses, and the point-biserial correlation between the overall number of changes and the presence of an event boundary. Within clauses, we found no association between the presence of an event boundary and changes in one of these dimensions (all $\phi < 0$) or the absolute number of changes ($r_{pbis} = -0.120$, $p = 0.145$); a clause that was associated with an event boundary was numerically even less likely to contain changes. We did, however, find

a significant association between the presence of an event boundary and the overall number of changes in the subsequent clause ($r_{pbis} = 0.295$, $p < 0.001$). This overall number of upcoming changes was a better predictor than most of the individual predictors; the only individual predictor that was even numerically better than the summed number of changes was time (time: $\phi = 0.305$, $p < 0.001$, space: $\phi = 0.271$, $p = 0.001$, object: $\phi = 0.175$, $p = 0.0342$ character: $\phi = 0.273$, $p < 0.001$, cause: $\phi = -0.091$, $p = 0.274$, goal: $\phi = 0.156$, $p = 0.0595$); i.e. subsequent changes in time had the strongest association with marked event boundaries. We further identified a significant association between the duration of the subsequent pause that followed a clause and the presence of an event boundary within that clause ($r_{pbis} = 0.409$, $p < 0.001$). These data suggest that participants successfully identify transitions between coherent events in the story.

Multiple regression analysis of "clause properties" onto neural CPR time course Because of the association between the presence of an event boundary and upcoming changes in the subsequent clause, we next wanted to know whether such upcoming changes in the story could explain CPR effects in the neural data. To this end, we implemented the following multiple regression analysis: Based on the finding that changes in narrative properties in the subsequent clause were correlated with the perception of an event boundary in the current clause, we created a $(time * 7)$ design matrix at the sampling rate of the neural data $(100Hz)$ that was set to 0 if no event boundary was marked; at time points where event boundaries were marked (based on the second behavioral run), we set the value to 1 if a subsequent change in time (column 1), space (column 2), object (column 3) or character (column 4) occurred. Those were the changes that we had linked to event boundaries with $p < 0.05$. At time points of event boundaries, column 5 was further set to the total number of changes in the upcoming clause; column 6 simply noted whether an event boundary was present, i.e., it was set to 1 at time-points where an event boundary was present and to 0 otherwise. Column 7 was reserved for the intercept (a column of ones). This design matrix was now used to fit a multiple regression onto the neural time-course of CPR, at various shifts of the time axis ($-10s$ to $+10s$, compare cross-correlation analysis in Supplementary Fig. 4a). This multiple regression analysis has the potential to answer which properties explain the increased CPR in the vicinity of event boundaries; the results from this analysis are shown in Supplementary Fig. 7. The first result from this analysis is that the multiple regression roughly replicates what was found with the cross-correlation that uses the continuous time-course of agreement on event boundaries (Supplementary Fig. 4a, red line): Prediction performance (r-squared) peaked approximately 1590 ms before the event boundary (compare 1610ms in the original cross-correlation analysis). When assessing the beta-weights of the predictors (time, space, object, character, number of changes, and event boundary) at this peak, however, only "event boundary" displayed a beta value that did not include 0 in the confidence interval (0.0168 ± 0.007, $95\%CI$); all other predictors: time (-0.0057 ± 0.0157), space (-0.0045 ± 0.0210), object (-0.0044 ± 0.0228), character (0.0008 ± 0.0200), number of changes (-0.0035 ± 0.0112). Indeed, when omitting the predictor event boundary, the prediction performance dropped substantially (Supplementary Fig. 7, black line). These data suggest that, even if changes in the

subsequent clause are correlated with the detection of an event boundary, these predictors are not helpful in explaining CPR effects. This buttresses the explanatory usefulness of the construct "event boundary".

Analyses of low level features in the story Because of the apparent association between pauses and event boundaries, we next wanted to explore whether low level features of the story (notably silences) could explain some of the effects in our data: We explored loudness (Supplementary Fig. 6b) and pitch (Supplementary Fig. 6c) of the audio recording in relation to event boundaries and to the neural data. When we locked the time-course of loudness (LU) to the 19 event boundaries in the same way that we had previously locked neural predictive recall to event boundaries, we found a significant decrease in loudness near the moments when participants marked event boundaries with a trough at $359ms$ before the event boundary (Supplementary Fig. 8a). The association between event boundaries and changes in pitch did not survive multiple comparisons correction. To ensure that silences in the story cannot explain our findings of enhanced predictive recall and information flow at event boundaries, we repeated all neural analyses involving event boundaries, using troughs in loudness instead of annotated event boundaries. No increase in predictive recall in the vicinity of silence survived correction for multiple comparisons (Supplementary Fig. 8b; compare Fig. 4b), and we did not observe a significant increase in hippocampo-cortical connectivity at peaks in silence in the story (Supplementary Fig. 8c). In conclusion, participants mark event boundaries at moments that precede upcoming changes in the story. This is evidence that annotators' judgement of event boundaries reflects meaningful structure in the story. Event boundaries also coincide with pauses and silences. Silences in the story, however, do not result in the neural correlates of event boundaries that we observe in our data.

Supplementary Note 9: Distinction of predictive recall from neural adaptation

Distinct topographies of predictive recall and neural adaptation Neural adaptation is described in the literature as the decreased responsiveness of neurons to a repeated stimulus [e.g. 83]. On a priori grounds, decreased responsiveness should not produce a temporal advance in neural processing of the sort that we detected with Granger Causality. We can also approach this question empirically by looking for electrodes that show an adaptation effect (defined as a reduced response to auditory stimulation on the second run of listening) or sensory enhancement (defined as an increased response to auditory stimulation on the second run of listening), and showing that effects of neural predictive recall are distinct from such adaptation and enhancement effects. This can be accomplished by demonstrating that the effects are not correlated across electrodes (i.e., electrodes that express strong adaptation do not also express strong predictive recall). To get at these questions, we can measure the neural response to the audio stimulus within the Granger Causality framework. In this, we consider the prediction of neural activity from the audio envelope within the full model, and contrast it with the prediction of the audio envelope from the neural signal. Specifically, we expect the audio to predict the brain, but not vice versa (or at least to a lesser extent). Indeed, this contrast maps out auditory processing regions (Supplementary Fig. 10, middle). In the case of neural adaptation, we

would now expect that – on the second run of listening – the audio-envelope would be less predictive of the neural signal than on the first run, because of neural adaptation to the repeated stimulus. Alternatively, the audio-envelope could be more predictive of the neural signal on the second run (enhanced sensory processing). We address this question by comparing the F values from the full model between the runs (audio-to-brain on run 2 versus audio-to-brain on run 1) and find that, on the second run of listening there is a substantially weaker neural response to the incoming audio stimulus (neural adaptation/fatigue, (Supplementary Fig. 10, left)). We find no evidence for an enhanced processing of the audio. The crucial question is now if this neural adaptation could explain the effect of better prediction from run 2 to run 1 (Supplementary Fig. 10, right). To address this question, we correlated the topographies of the effects. The correlation across all electrodes ($N = 988$) between the predictive recall effect and the neural adaptation effect was $r(986) = 0.0035$ ($p = 0.912$, n.s., note: we would expect a negative correlation because the effects have opposite signs). The correlations (across electrodes) between the audio entrainment effect (audio-to-brain) and predictive recall, on the one hand, and audio entrainment and neural adaptation, on the other hand, were $r(986) = 0.389$ ($p < 0.001$) and $r(986) = -0.411$ ($p < 0.001$) respectively. These data are evidence that neural adaptation and predictive recall take place simultaneously in distinct subsets of electrodes that show a response to the auditory stimulation.

No relation of neural adaptation to event boundaries, behavioral prediction learning, or hippocampo-cortical interactions We next wanted to extend the investigation of neural adaptation further, because it provides a convenient control analysis for the main claims in our manuscript (i.e., we would not expect any of the behavioral or neural correlates of predictive recall to occur with adaptation). To this end, we built a time-course of neural adaptation in the same way that we built the neural time-course of predictive recall. First, we identified electrodes that expressed neural adaptation (30 channels across 5 subjects) via Gaussian Mixture Modelling. We then projected the model predictions at these electrodes (audio envelope predicting the neural signal) onto the neural signal from the respective run and subtracted run 1 from run 2. In this time-course, lower values reflect more adaptation. When we locked this time-course to event boundaries (compare to Fig. 4b), we did not observe more adaptation in the vicinity of event boundaries (all uncorrected $ps > 0.091$, one-sided). We next correlated the time-course of neural adaptation with the change in prediction probability (compare Fig. 4c). At no lag did this correlation survive the correction for multiple comparisons. Notably, the strongest correlations between the word-prediction learning and the time course of adaptation were in the positive direction (we would however expect negative correlations, because more adaptation results in more negative values) and were observed at a negative shift (i.e., before word onset). At no lag was the correlation below correlations from random permutation of word-prediction-changes (experiment: all uncorrected $ps > 0.19$, replication: all uncorrected $ps > 0.69$). We next computed mutual information between hippocampus and neural adaptation electrodes in the 5 subjects that had hippocampal and neural-adaptation-channels. Therein, we performed the analysis of mutual

information in the vicinity of individual peaks in neural adaptation (local minima in the time-course). When contrasting the second time of listening with the first time of listening, we did not observe any time-point that displayed enhanced hippocampo-cortical interaction on the second time of listening (all uncorrected $ps > 0.06$, one-sided).

Supplementary Note 10: Control analyses confirming earlier boundary detection on the second run of listening

In the behavioral boundary detection experiment, we wanted to confirm that a sharpening in response profiles could not lead to a spurious finding of a shift in the cross-correlation between the runs. In order to simulate such a sharpening in our data – where peaks become more pronounced and values outside of the peak become more attenuated – we repeated the cross-correlation analysis between the agreement on run 1 and the squared agreement on run 2. Despite substantially sharpening the response profile, this manipulation resulted in a peak of the cross-correlogram at a lag that was even slightly less pronounced (-172ms). A sharpening of responses can therefore not explain the negative lag at the peak in cross-correlation that we find. We next set out to investigate the change in response in more detail. In order to illustrate the distribution of responses, we locked the responses from run 1 and from run 2 to the event boundaries that were defined on run 2, summed across participants and averaged across all boundaries (Supplementary Fig. 11, left). The agreement on run 2 displayed an earlier increase and the peaks of the curves were $141ms$ apart in time (i.e., peaked earlier on run 2). We then randomly shuffled the labels of run 1 and run 2, 1000 times for each subject and computed the temporal difference in peaks. Out of these 1000 shuffles, the random run 2 peaked more than $141ms$ before the randomly defined run 1 only 37 times ($p = 0.037$). In order to not bias the results by selecting the event boundaries from run 2, we repeated the same analysis using 18 event boundaries that were defined on run 1. Again the time course of agreement on the second run started to rise earlier and peaked $196ms$ earlier (Supplementary Fig. 11, right) than the time-course of agreement from run 1 ($p = 0.014$ from 1000 random permutations), i.e., peaks in agreement on the second run are significantly earlier than on the first run, independent of whether the agreement is time-locked to event boundaries defined on the first or second run of boundary norming. Finally, we ran another variant of the analysis where we derived new event boundaries on the same subjects with each random permutation. Defining boundaries based on the intact run 2 and on the intact run 1 also yielded a higher difference in peak-times than under 1000 random permutations with re-defined boundaries ($p = 0.012$ and $p = 0.001$, respectively).

Supplementary Note 11: Analysis of potential backwards association

In addition to looking for electrodes that showed a positive delta-F effect (indicating predictive recall), we also looked for electrodes that showed a negative delta-F effect. While there was no overall main effect in the negative direction, it might be possible that the positive delta-F-values dominate the average delta-F and thereby mask an effect in the opposite direction. Such an effect could, for instance, reflect backwards

association where moments from the first run of listening appear slightly later in the second run because they are remembered more. To investigate this hypothesis, we first removed all electrodes that were identified as CPR electrodes and then tested the remaining electrodes for a main effect in the negative direction. We repeated the same statistic of electrode-wise and subject-wise permutation, however, we did not find a main effect in the negative direction ($ps > 0.097$, compare to Supplementary Fig. 3a). Nonetheless, we wanted to test whether some electrodes could have meaningful signal that captures, e.g., backwards association in the story. To accomplish this, we used the negative of the threshold that was used to identify CPR electrodes in order to identify electrodes that showed negative delta-F values: a total of 3 channels distributed across 2 patients had delta-F values that fell just slightly below that negative threshold. For these channels we built a time-course in the same way as we did in the CPR analysis (mean projected model difference, compare to Fig. 4a), where we are now interested in negative values. In a first analysis, we locked this time-course to event boundaries (compare to Fig. 4b, however, no significant decrease was observed. We next correlated this new time-course with the word-prediction learning from the behavioral experiment (compare to Fig. 4c); however, no significant correlation was observed at any of the lags, either in the first experiment (all $p > 0.36$) or in the replication (all $p > 0.166$). Finally, we analysed mutual information between hippocampus and the identified electrodes in the 2 patients (they both had hippocampal electrodes). We did not observe a significant increase in MI on the second run of listening (compare to Fig. 5a-b). In conclusion, despite potentially interesting implications, identifying electrodes with (non-significant) negative delta-F values did not yield any effects in our other analyses.

Supplementary Note 12: Exploratory analysis of neural predictive recall at different frequencies and sampling rates

In order to explore the idea that other frequency bands may display similar CPR effects with different choices for sampling rate, we repeated the Granger Causality analysis for a range of sampling rates that still permits an analysis with a reasonably high temporal resolution: We analysed every frequency band at a sampling rate of 10, 15 and $20Hz$ and between $25Hz$ and $200Hz$ in steps of $25Hz$ and repeated the electrode-wise permutation statistic (now with 20000 permutations to obtain sufficient precision on p-values). When we corrected for multiple comparisons by controlling the false discovery rate, we found that the high-gamma frequency-band showed the same GC main effect for all of these sampling rates ($p_{FDR} = 0.0101$). Interestingly, at sampling-rates lower than $25Hz$ (i.e., at a resolution of maximally $40ms$) we found a significant main effect for the low gamma band ($ps < 0.001$). At 20 and $25Hz$ we further observed a significant main effect in the alpha frequency band ($ps <= 0.005$), i.e., while we found the high-gamma effect to be the most robust and not to be susceptible to different choices of sampling rate, the alpha and the low-gamma frequency band could benefit from different and substantially lower sampling rates. We next analyzed the low gamma and alpha frequency band at the sampling rate that resulted in the biggest average difference in between-run prediction ($10Hz$ for low gamma and $20Hz$ for alpha). When we tried to

separate channels that show a CPR effect from channels that didn't show an effect, however, the distribution of $\Delta - F$ values appeared less separable: Fitting the Gaussian Mixture Model did not always converge and the parameters of the 2 distributions were unstable under different iterations. In order to threshold electrodes that could be considered CPR electrodes in the alpha and low gamma band, we therefore z-scored the $\Delta - F$ values and only considered channels above z-scores corresponding to the 99th percentile of the normal distribution. Thereby we identified 16 channels for the low gamma frequency band and 11 channels for the alpha frequency band (Supplementary Fig. 14a-b). We then built the time-courses of predictive recall in the same way as for the high gamma frequency band, however, we did not observe any significant correlation of the time-courses with word prediction learning or increased predictive recall near event boundaries in the alpha frequency band. The low gamma frequency band only displayed a significant correlation with word-prediction learning in the replication study ($p_{FDR} = 0.016$, but not in the original experiment). This correlation, however, was significantly increased at all lags between $1.9s$ prior to word onset to $2s$ past word onset (at a sampling rate of $10Hz$ i.e. there were 41 tested lags) and did not express a clear peak; i.e., interpretation of this effect is limited due to the low temporal resolution. To ensure that the lack of signal was not due to the number of electrodes that we selected, we next repeated these analyses with the 31 channels with the strongest CPR effect, however, we observed the same results ($p_{FDR} = 0.0240$ only for the behavioral prediction replication correlated with the low-gamma CPR). In an exploratory analysis we further wanted to test, whether the lack of effects could be due to a loss of signal due to the low sampling rate. We therefore scrutinized effects in the high gamma ($70 - 200Hz$) frequency band at different sampling rates. Indeed, when we repeated the key analyses of CPR relative to event boundaries and CPR correlation with behavioral prediction learning, these effects are only evident at sampling rates of $20Hz$ or higher. For this reason, we repeated the analysis of the alpha and low gamma frequency band at the highest significant sampling rate (i.e., $25Hz$). We observed numerically increased correlation with word-prediction learning for the alpha and low gamma band (17 CPR channels each selected via z-score) that did not survive correction for multiple comparisons. In conclusion, the high gamma frequency band displays the main effect and the relation to word-prediction learning and event boundaries robustly for different choices of sampling rate, however, analyses that hinge on fine grained temporal resolution of the CPR time-course need a sampling rate of at least $25Hz$. The alpha and low gamma frequency band both contain significant CPR signal and can benefit from a lower sampling rate, however, the signal-to-noise ratio in these data is lower and the reduced temporal resolution that comes with the lower sampling rate does not allow for fine-grained analyses of predictive recall.

Supplementary Figures

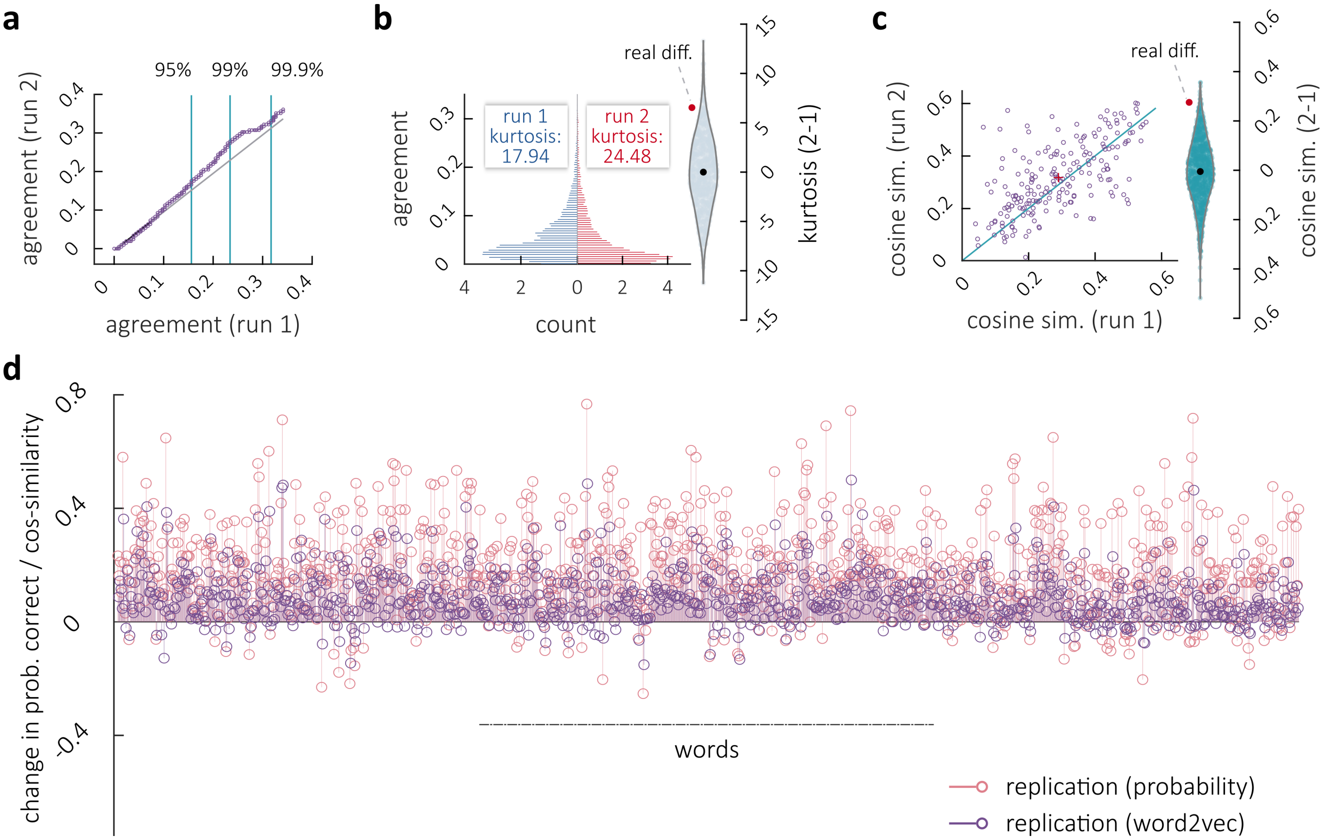

Supplementary Figure 1 | Behavioral analyses demonstrating predictive recall. a. Quantile-quantile plot comparing the distribution (across time) of agreement on event boundaries (compare Fig. 1a) between runs. The deviation from a diagonal line indicates a relative over-representation of moments of high agreement on the second run. **b.** Histograms depicting the distribution (across time) of agreement on the first run (left, blue) and on the second run (right, red). Higher kurtosis on the second run indicates that more values fall in the tails of the distribution. The violin plot displays the run-differences in kurtosis under random label permutation (gray) and the true difference (red dot) **c.** Similarity of each subject's response vector to the agreement across all others (same as Fig. 1b). Purple dots indicate individuals: subjects above the diagonal increase in similarity to others on the second run. The average increase (red cross) indicates consensus learning. The violin plot displays the average run-difference in cosine similarity to others under random label permutation (turquoise) and the true difference (red dot). Source data are provided as a Source Data file. **d.** Performance difference between groups that had listened to the story and naive participants in prediction-measures for upcoming words in the story. Difference in prediction-probability (fuchsia replication) and cosine-similarity between predicted and correct word (purple experiment 2) are higher after a single exposure.

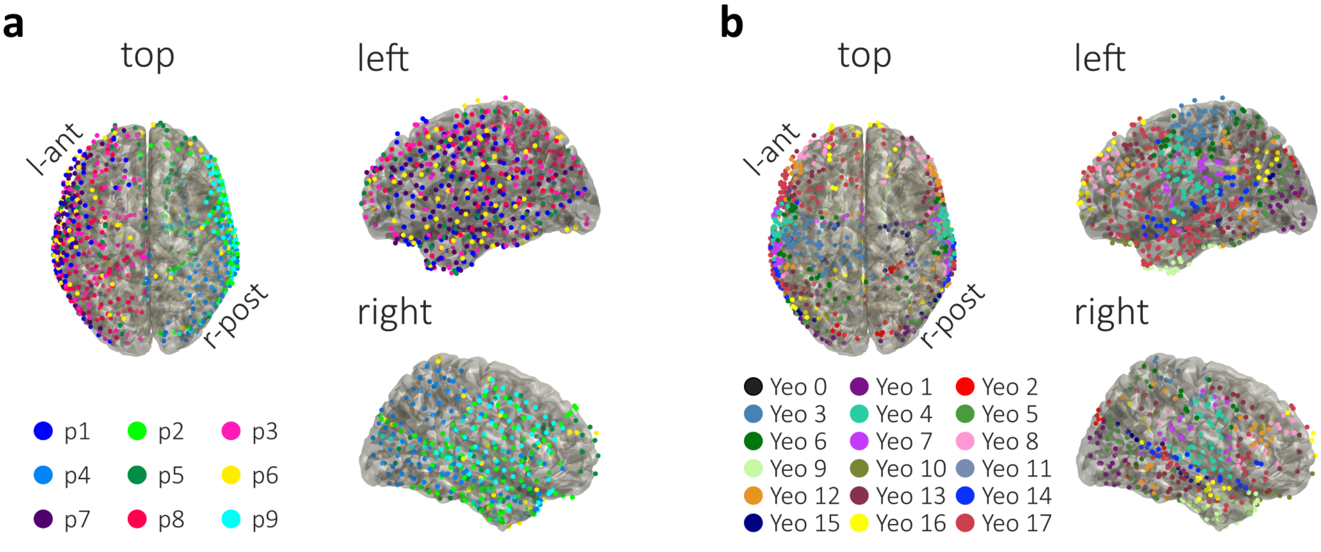

Supplementary Figure 2 | Electrode placement and anatomical labels. **a.** Electrode placement across nine patients showing extensive coverage across both hemispheres. **b.** Anatomical labels of electrodes according to the yeo 17 network solution (see: Online Methods).

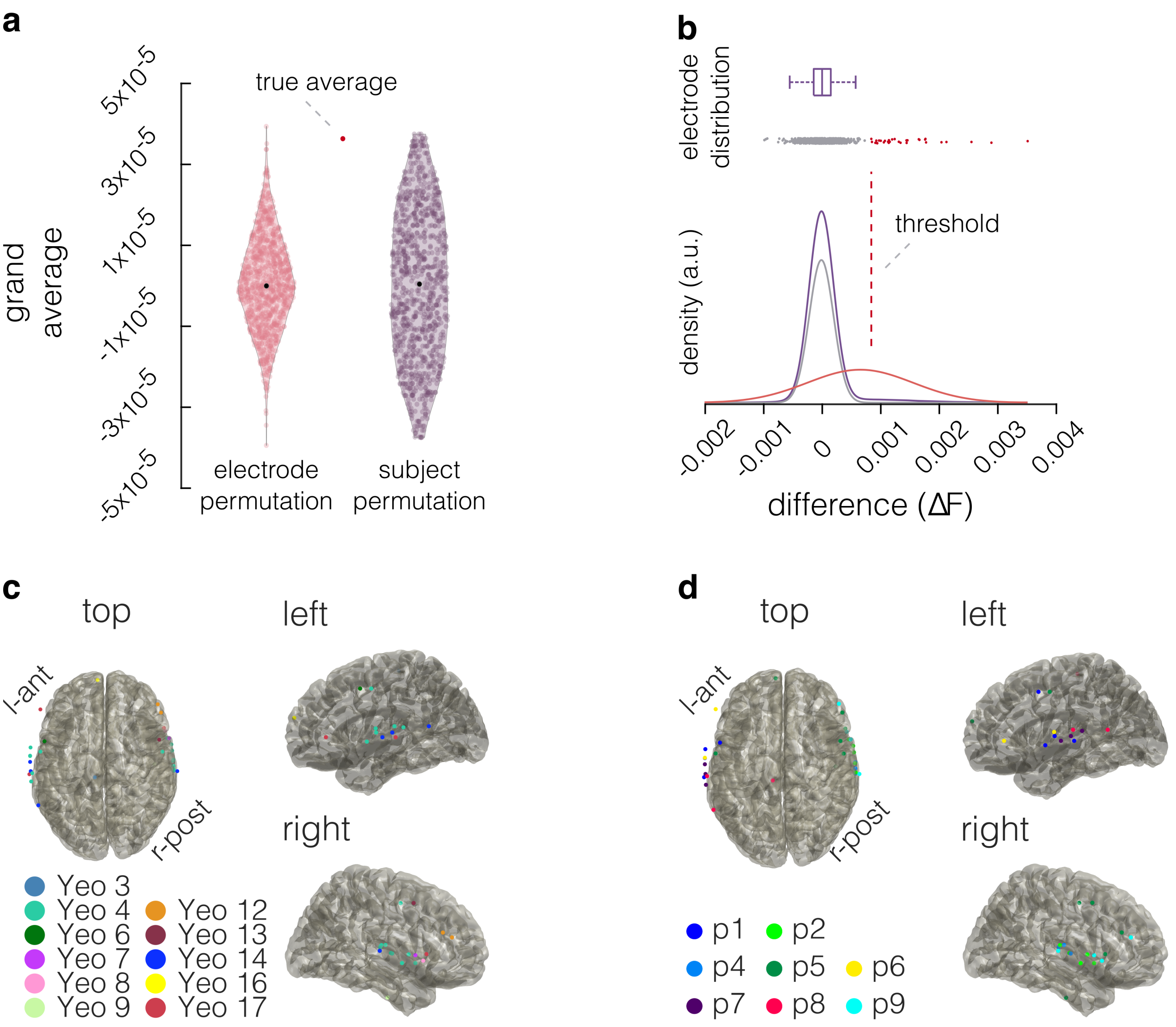

Supplementary Figure 3 | Statistics of neural predictive recall, electrode selection and labeling for 'CPR-channels'. a. Differences in F-values measuring predictive recall (from the Granger Causality analysis) were averaged across all channels (true average, red dot). The pink violin plot displays these average values under random assignment of run-labels per channel. The purple violin plot displays average values under random assignment per subject (compare Fig. 2). **b.** Gaussian-Mixture-Modeling of the distribution of differences with 2 Gaussian distributions. Electrodes were selected as displaying predictive recall if they were 10 times more likely to belong to the distribution with the higher mean. The mixed distribution is purple, the gray Gaussian represents the null-distribution and the red Gaussian the effect-distribution (scaled to an AUC of 1 for visibility). Dots are electrodes in the color of their assigned distribution. Source data are provided as a Source Data file. **c.** Effect-electrodes are colored according to their position in the 17 network parcellation of the Yeo-atlas. **d.** Effect-electrodes are colored according to the patient from which they stem.

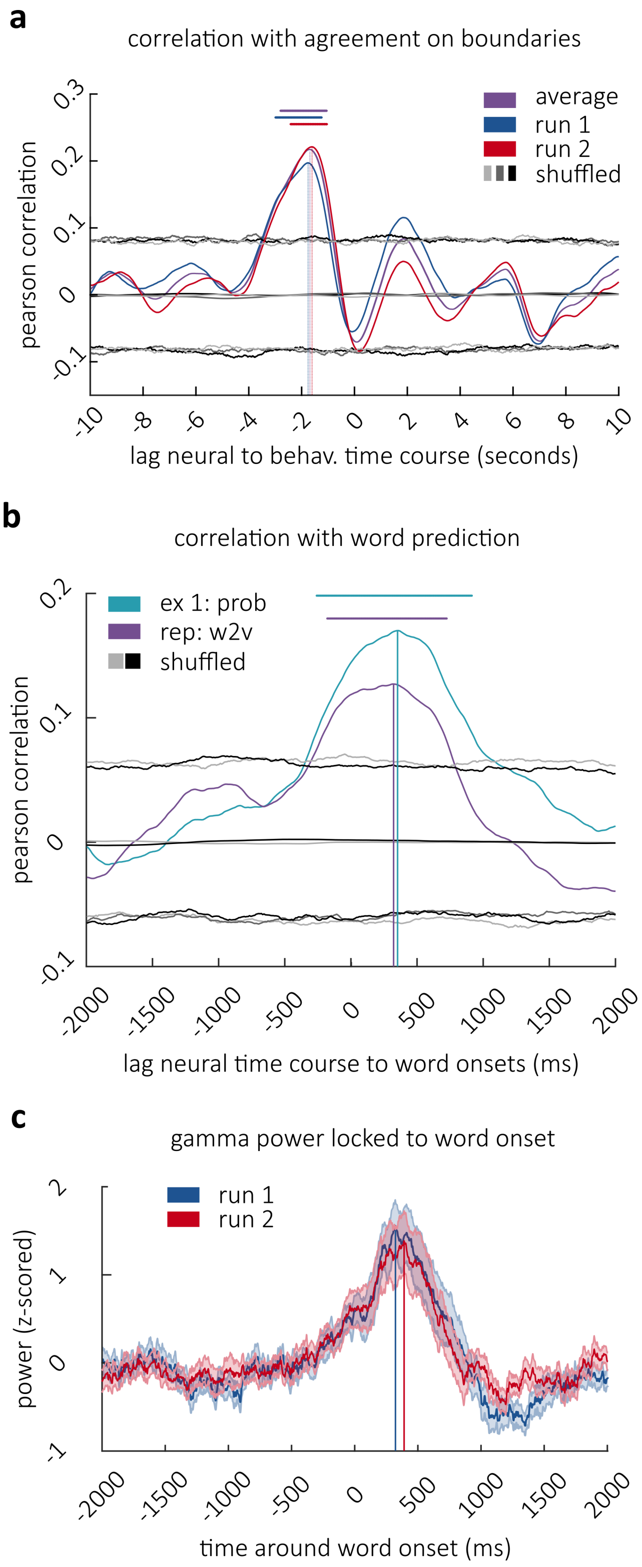

Supplementary Figure 4

Supplementary Figure 4 | Cross-correlation between neural prediction and event boundary time-course and gamma response to word onset on CPR-channels. a. Cross-correlograms of the neural time-course of predictive recall with the time-course of agreement on boundaries (compare Fig. 1a, with average between behavioral runs: purple, with behavioral run 1: blue, with run 2: red), vertical lines mark maxima. Gray-scaled lines are 5th and 95th percentile of cross-correlations with phase-shuffled neural data. Horizontal lines mark significance (fdr-corrected). **b.** Correlation between the behavioral measures of word-prediction learning (compare Fig. 1e, turquoise, Supplementary Fig. 1e, purple) and the neural time-course of predictive recall at different time-lags. Line in turquoise shows correlation between the probability of correct prediction in experiment 1, the purple line represents the correlation with the difference in cosine-similarity from the replication, which is arguably a more sensitive measure of prediction learning. Gray lines are 5th and 95th percentile of correlations under random assignment of performance to words for the respective measures. Horizontal lines mark significance (as per false discovery rate correction average: $p_{FDR} = 0.002$, run 1: $p_{FDR} = 0.002$, run 2: $p_{FDR} = 0.001$), vertical lines mark peaks. **c.** Average response $(+-SEM)$ in the gamma band to word onset in run 1 (blue line) and run 2 (red line) on CPR-channels. Vertical lines mark peaks.

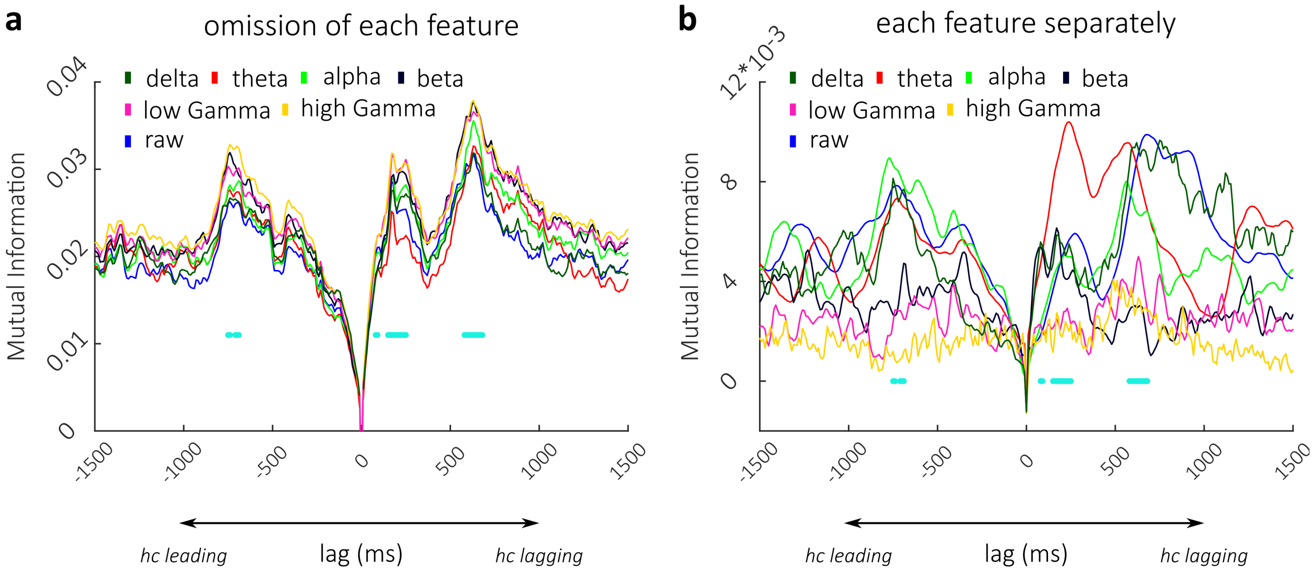

Supplementary Figure 5 | Feature by feature re-analysis of hippocampo-cortical connectivity at moments of predictive recall on run 2. a. Conditional Multivariate Mutual Information at different lead/lag to CPR-channels at neural predictive recall events was re-analyzed on run 2 omitting each hippocampal feature once. These data show the contribution of features to MI via reduction in MI. **b.** Conditional Multivariate Mutual Information at different lead/lag to CPR-channels at neural prediction events was re-analyzed on run 2 using each hippocampal feature on its own. These data show the contribution of features to MI, specifically, how much MI is obtained by using each feature on its own. Horizontal lines depict points of significance from Fig. 5a, right in turquoise.

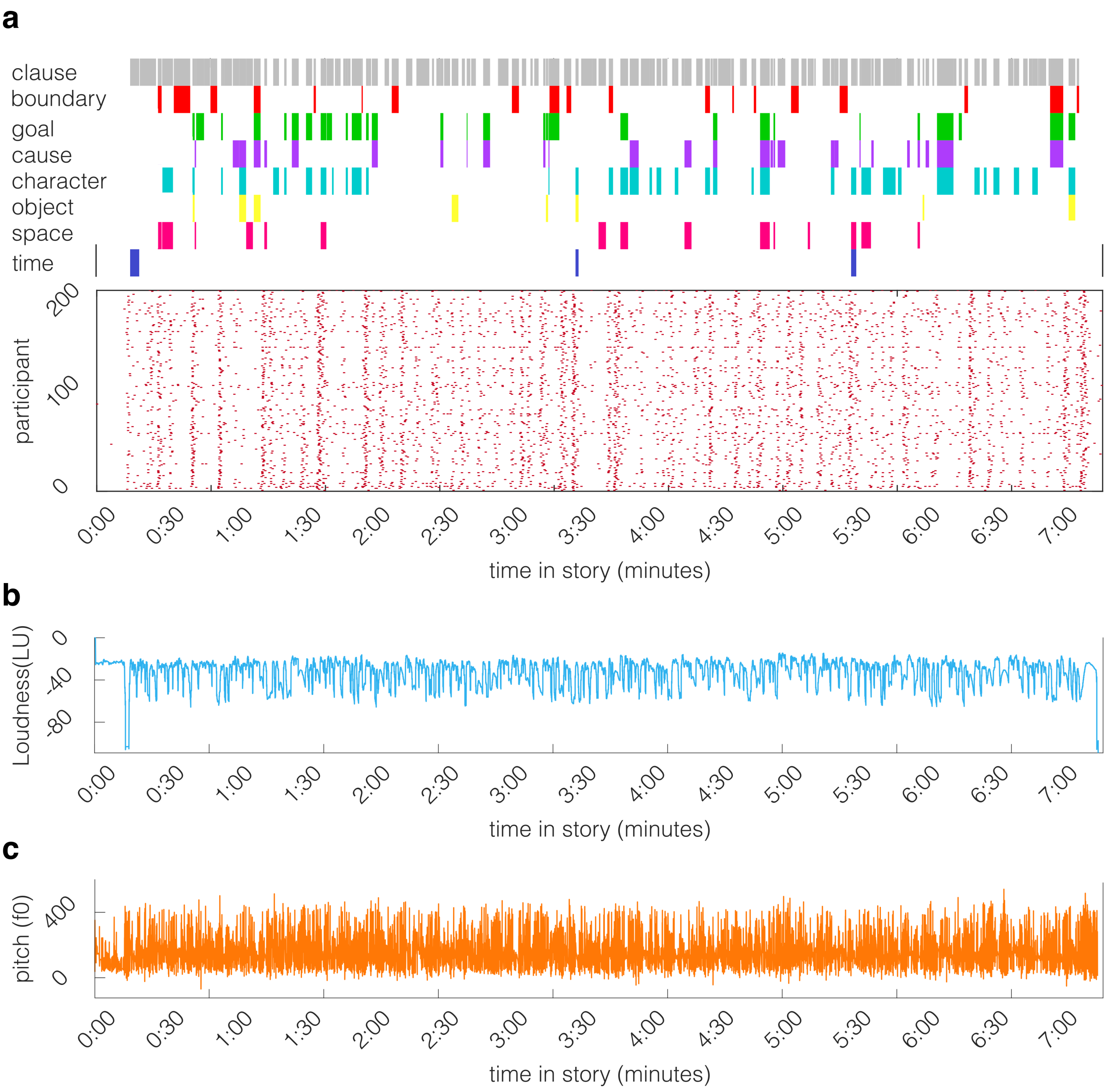

Supplementary Figure 6 | Characterization of event boundaries in the story. a. Clauses marked according to whether a particular property changed and whether an event boundary occurred in the clause (top row, colored clauses reflect that the property is present), and individual participants' responses as a raster plot (bottom row). Event boundaries were more likely to occur if the subsequent clause contained more changes (overall) or a change in time, space, object or character (a statistical trend was observed for changes in goal). **b.** Time course of loudness (Loudness Units) for the story. **c.** Time course of pitch (fundamental frequency) throughout the story.

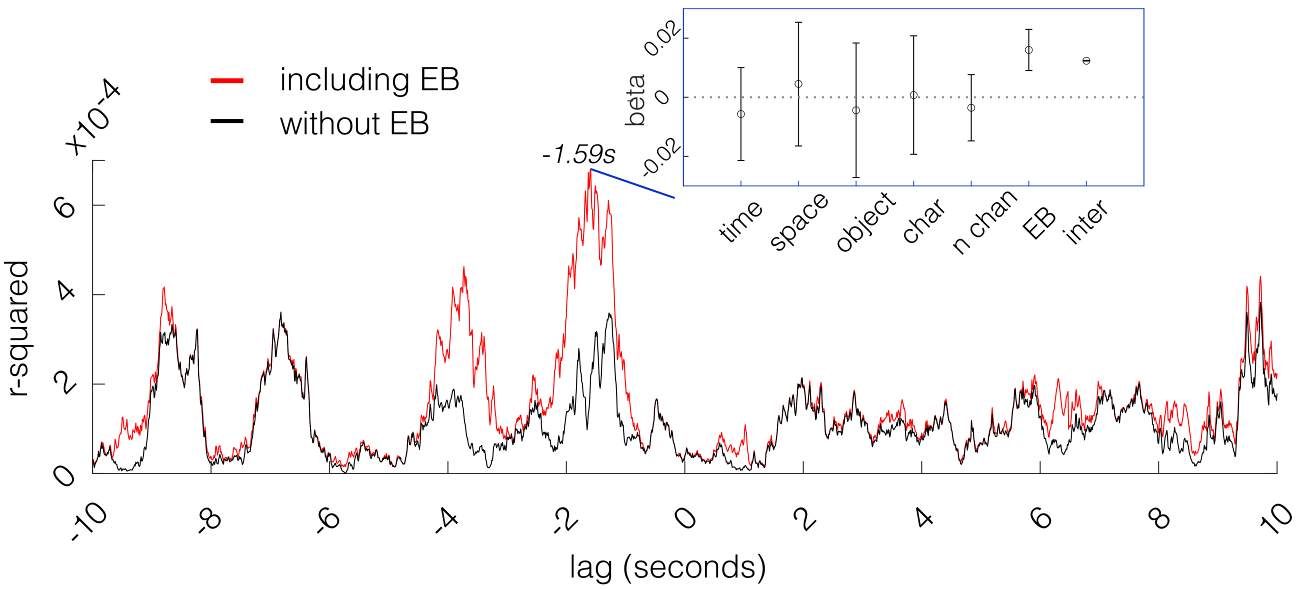

Supplementary Figure 7 | Multiple regression of "clause properties" that are associated with event boundaries onto the neural CPR time course. Approximately 1.59s before event boundaries, the regression onto the neural signal peaks (compare to Supplementary Fig. 4a, red line for a cross-correlation with the time-course of agreement). The inset displays beta values of predictors at the peak (whiskers are 95%CI, the dot denotes the beta estimate). At the prediction-peak, only the predictor Event Boundary (EB) has a beta value that doesn't include 0 in the confidence interval. All other predictors (upcoming change in time, space, object, character, and absolute number of upcoming changes) have CIs that include 0.

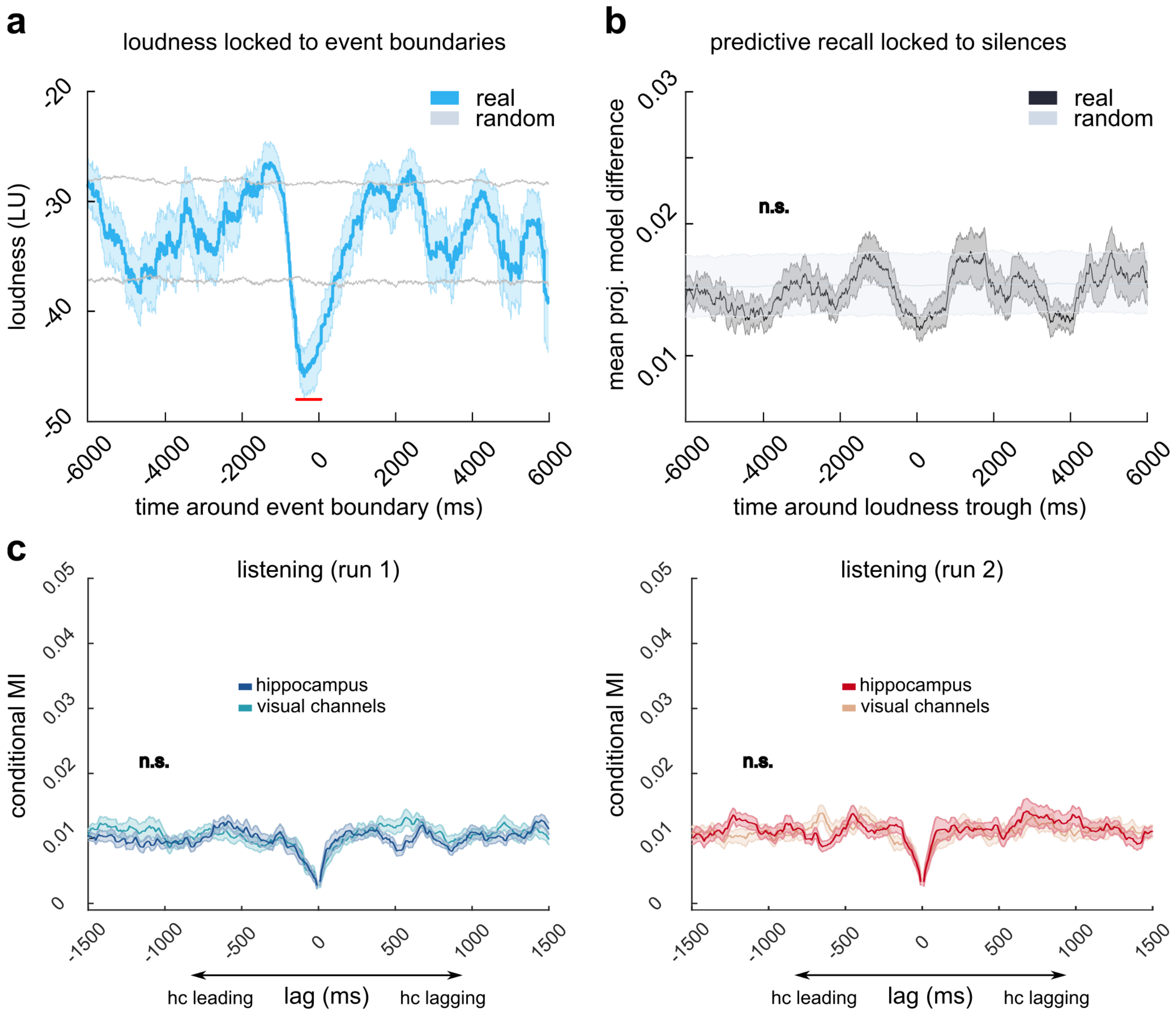

Supplementary Figure 8 | Analysis of silences in the story. a. Loudness of the audio is significantly lower in the vicinity of event boundaries. Error bars represent SEM around the mean across 19 event boundaries, red dots represent time-points of significance (as per 1000 random assignments of event boundaries $p_{FDR} = 0.002$) **b.** Time course of neural predictive recall, locked to silences in the audio. Despite their correlation with event boundaries, silences in the story are not associated with increased neural predictive recall. Error bars (dark gray) represent SEM around the mean for the real data; light gray bands show the 5th and 95th percentile for data derived from random moments in the story. **c.** Connectivity between hippocampus and CPR channels at silences. There is no significant increase in hippocampo-cortical connectivity at troughs in loudness, when compared to a visual control region. Error bars represent SEM around the mean across electrodes. The scaling of the y-axis is according to the corresponding analyses in the manuscript.

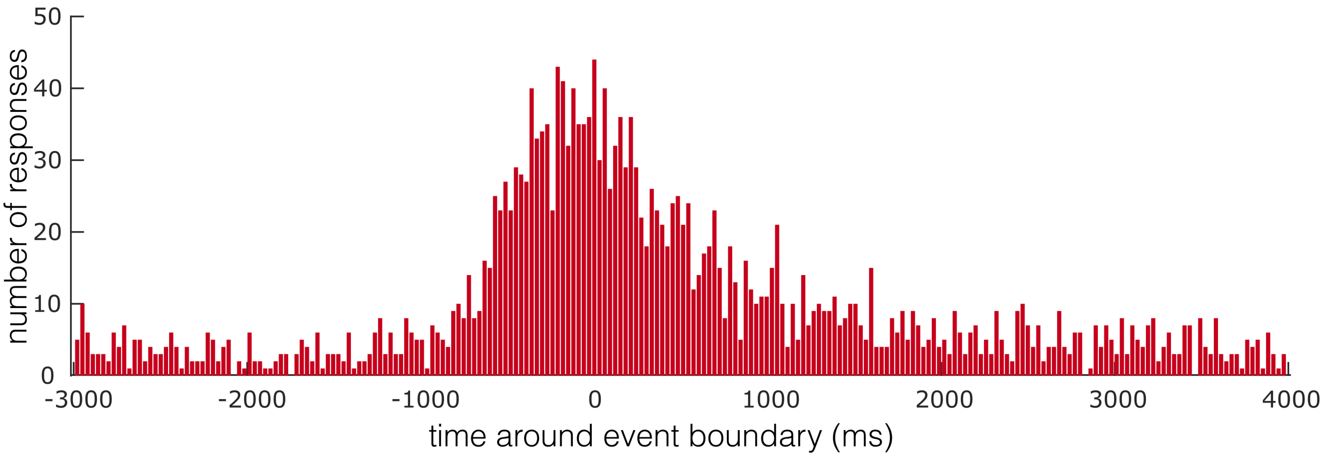

Supplementary Figure 9 | Histogram of response times around 19 event boundaries on second behavioral run. The histogram displays an ex-gaussian distribution of responses with a single peak in the center, consistent with what we would observe if there were variable RTs to a single event boundary. A substantial part of this distribution falls outside of the 1-second window around zero (+-500ms), suggesting that relying on this 1-second window to compute agreement might underestimate the number of participants who noticed a particular boundary.

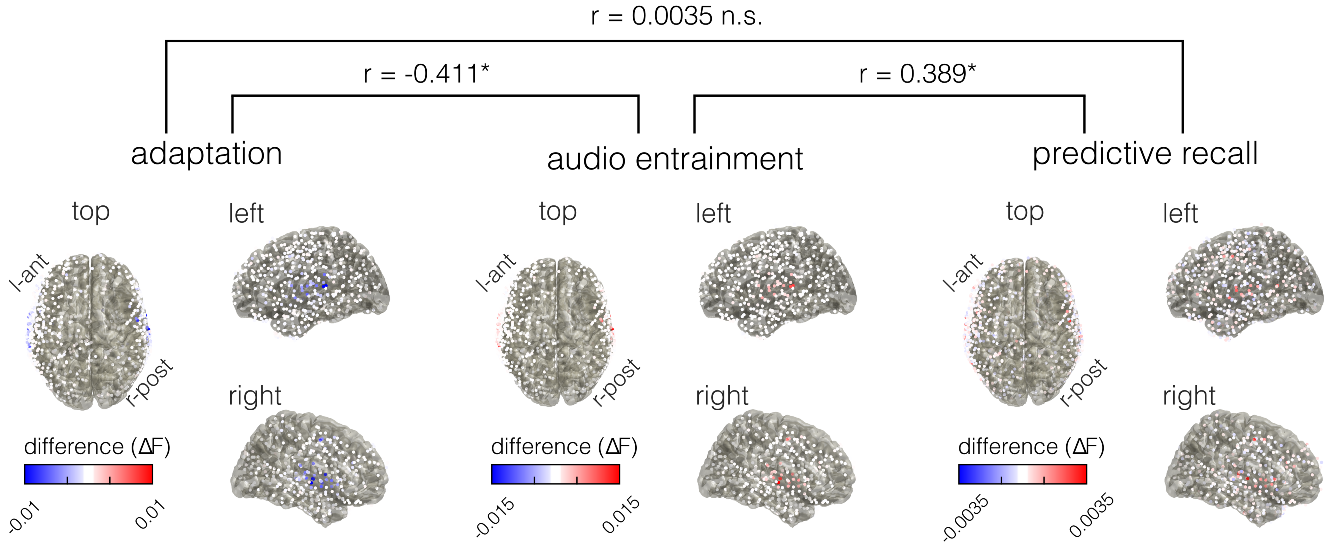

Supplementary Figure 10 | Neural adaptation vs. predictive recall. On the second run of listening, the audio envelope becomes less predictive of the neural signal (left), which can be interpreted as neural adaptation, i.e. a weaker response to a stimulus. This map is correlated with the map of audio entrainment (middle, where the audio envelope is predictive of neural gamma band activity). Predictive recall (right) is also correlated with the map of audio entrainment, however, there is no significant correlation between neural adaptation and predictive recall.

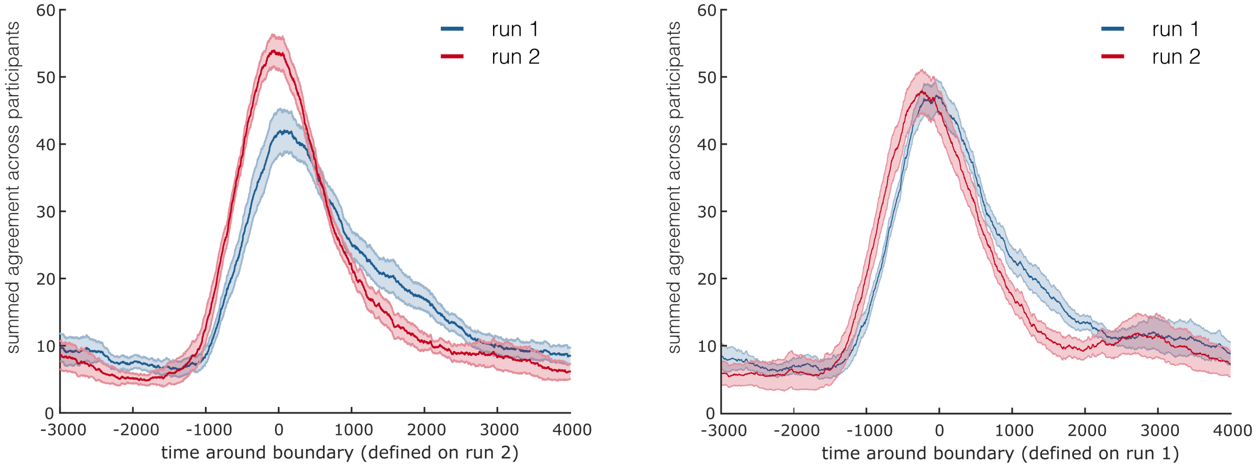

Supplementary Figure 11 | Time course of agreement locked to event boundaries. Event boundaries could either be defined based on the second run (left) or the first run (right) of listening. In both cases the summed agreement across participants was averaged across the event boundaries. Lines display averages $\pm SEM$. An earlier rise and peak are visible in the time-course of agreement on the second run (as confirmed by permutation statistics, see: Supplementary Text).

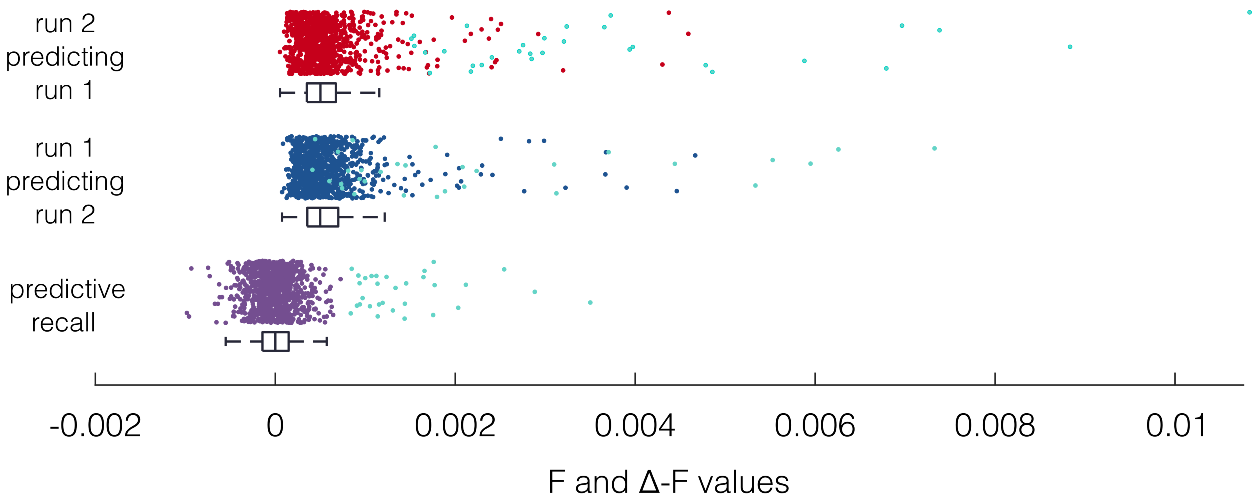

Supplementary Figure 12 | Distribution of F-values and their difference. Distribution of F-values that capture the prediction from run 2 to run 1 (top row) and from run 1 to run 2 (middle row). Their difference is displayed in the bottom row. Channels that exceed the threshold for CPR channels are highlighted in turquoise.

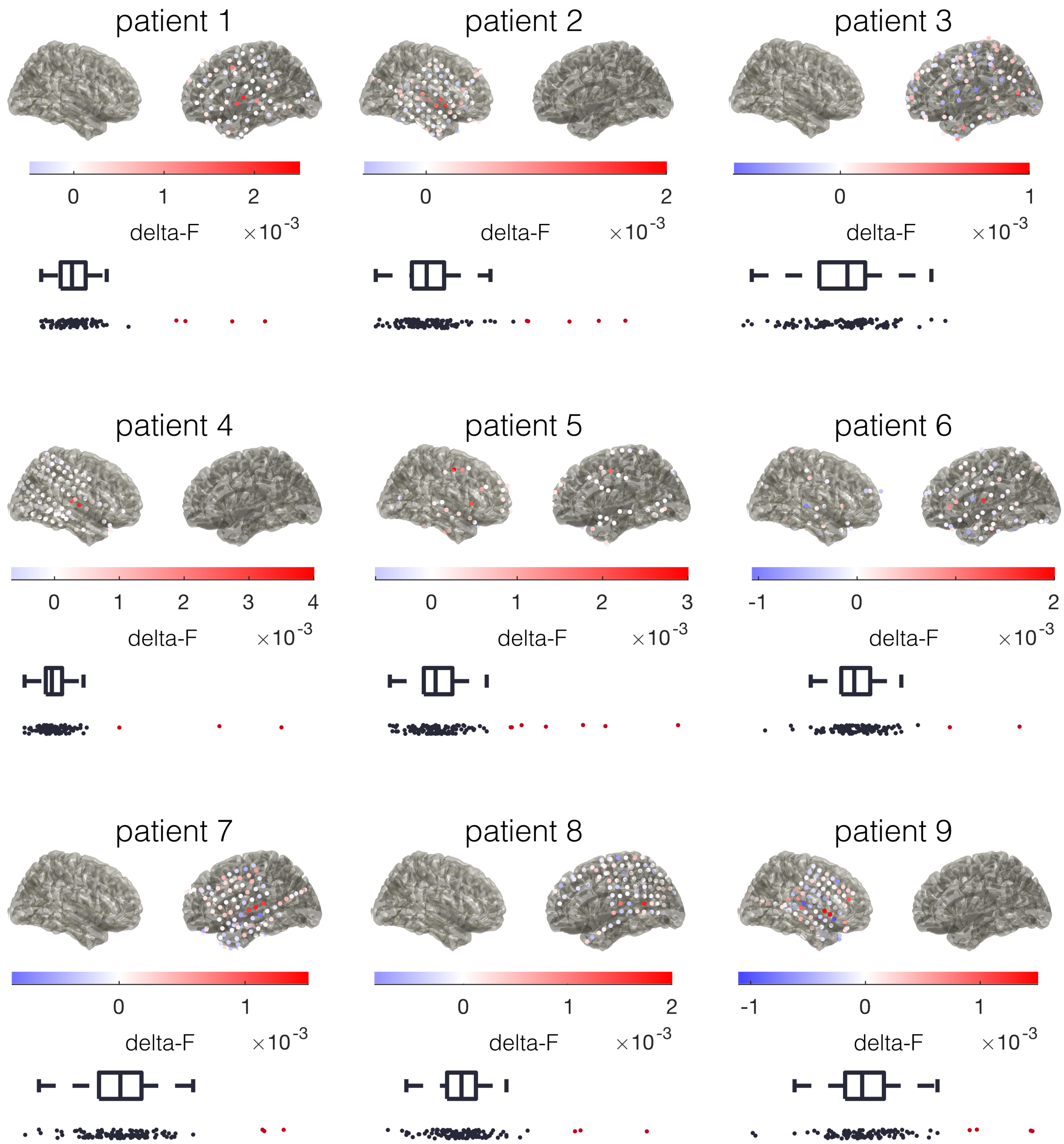

Supplementary Figure 13 | Distribution of differences in F-values across patients. The difference in F-values is displayed for individual patients at MNI positions (top) or as a distribution (bottom). Color-bars are symmetric, but truncated for visibility. Boxplots share the x-axis with the colorbar and display 25th and 75th percentile around the median, whiskers are the most extreme data points not considered outliers (as per 2.5 interquartile ranges above/below the median). Black dots below the boxplot are individual electrodes. Electrodes where cortical predictive recall was identified are highlighted as red dots. Note that only patient 3 did not have any CPR-electrodes. The number of displayed electrodes are 92, 117, 113, 117, 118, 111, 107, 109, and 104. Source data are provided as a Source Data file.

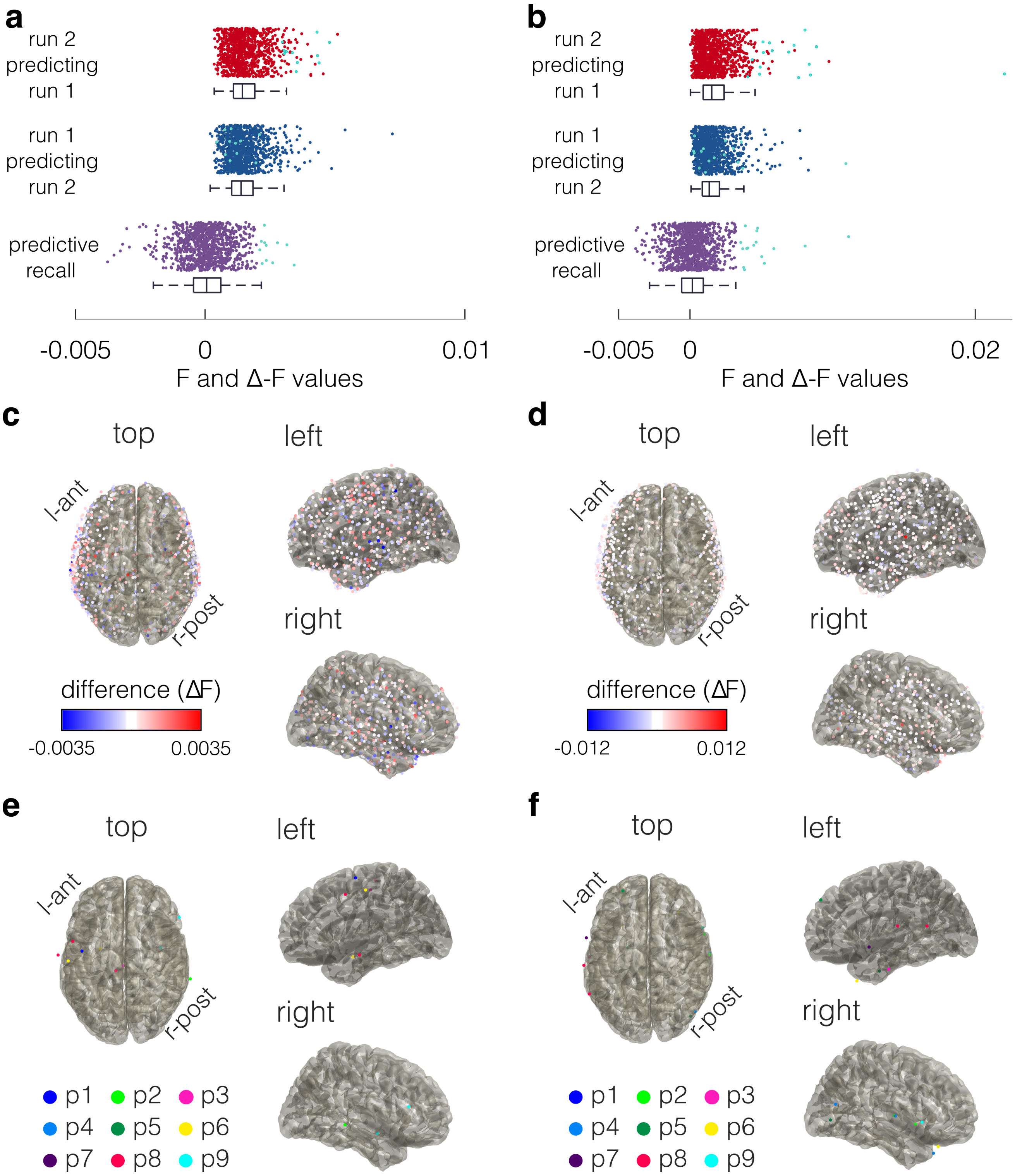

Supplementary Figure 14

Supplementary Figure 14 | Exploratory analyses of neural predictive recall at different frequency bands and sampling rates. a. Distribution of F-values that capture the prediction from run 2 to run 1 (top row) and from run 1 to run 2 (middle row) and their difference (bottom row) in the alpha band at a sampling rate of $20Hz$ and **b.** in the low gamma band at a sampling rate of $10Hz$. Channels that exceed the threshold for CPR channels are highlighted in turquoise. **c.** Difference in F-values between the prediction of run 1 from run 2 and the prediction of run 2 from run 1, indicating neural evidence for predictive recall in the alpha band at a sampling rate of $20Hz$ and **d.** in the low gamma band at a sampling rate of $10Hz$. **e.** Distribution of CPR channels across patients in the alpha band at a sampling rate of $20Hz$ and **f.** in the low gamma band at a sampling rate of $10Hz$.

Supplementary Table 1

Details on channel count

Patient	chan included	chan exluded	ICA comp excluded	n HC chan	n CPR chan
1	92	3	1	0	4
2	117	1	1	2	5
3	113	8	1	0	0
4	117	0	3	4	3
5	118	4	2	4	7
6	111	9	3	6	2
7	107	4	1	6	3
8	109	15	2	0	3
9	104	0	1	8	4

References

[1] Chen, J. *et al.* Shared memories reveal shared structure in neural activity across individuals. *Nat. Neurosci.* **20**, 115–125 (2017).

[2] Lee, H., Bellana, B. & Chen, J. What can narratives tell us about the neural bases of human memory? *Curr. Opin. Behav. Sci.* **32**, 111–119 (2020).

[3] Baldassano, C. *et al.* Discovering event structure in continuous narrative perception and memory. *Neuron* **95**, 709–721.e5 (2017).

[4] Kauttonen, J., Hlushchuk, Y., Jääskeläinen, I. P. & Tikka, P. Brain mechanisms underlying cue-based memorizing during free viewing of movie Memento. *NeuroImage* **172**, 313–325 (2018).

[5] Sonkusare, S., Breakspear, M. & Guo, C. Naturalistic stimuli in neuroscience: critically acclaimed. *Trends Cogn. Sci.* **23**, 699–714 (2019).

[6] Bartlett, F. C. *Remembering: A study in experimental and social psychology* (Cambridge University Press, New York, NY, US, 1932).

[7] Zacks, J. M., Speer, N. K., Swallow, K. M., Braver, T. S. & Reynolds, J. R. Event perception: A mind-brain perspective. *Psychol. Bull.* **133**, 273–293 (2007).

[8] Radvansky, G. A. & Zacks, J. M. Event boundaries in memory and cognition. *Curr. Opin. Behav. Sci.* **17**, 133–140 (2017).

[9] Zacks, J. M., Speer, N. K. & Reynolds, J. R. Segmentation in reading and film comprehension. *J. Exp. Psychol. Gen.* **138**, 307–327 (2009).

[10] Pettijohn, K. A. & Radvansky, G. A. Walking through doorways causes forgetting: environmental effects. *J. Cogn. Psychol.* **28**, 329–340 (2016).

[11] Lawrence, Z. & Peterson, D. Mentally walking through doorways causes forgetting: The location updating effect and imagination. *Mem. (Hove, England)* **24**, 12–20 (2016).

[12] Reynolds, J. R., Zacks, J. M. & Braver, T. S. A Computational Model of Event Segmentation From Perceptual Prediction. *Cogn. Sci.* **31**, 613–643 (2007).

[13] Franklin, N. T., Norman, K. A., Ranganath, C., Zacks, J. M. & Gershman, S. J. Structured event memory: A neuro-symbolic model of event cognition. *Psychol. Rev.* **127**, 327–361 (2020).

[14] Newtson, D. Attribution and the unit of perception of ongoing behavior. *J. Pers. Soc. Psychol.* **28**, 28–38 (1973).

[15] Speer, N. K., Zacks, J. M. & Reynolds, J. R. Human Brain Activity Time-Locked to Narrative Event Boundaries. *Psychol. Sci.* **18**, 449–455 (2007).

[16] Whitney, C. *et al.* Neural correlates of narrative shifts during auditory story comprehension. *NeuroImage* **47**, 360–366 (2009).

[17] Zacks, J. M. *et al.* Human brain activity time-locked to perceptual event boundaries. *Nat. Neurosci.* **4**, 651–655 (2001).

[18] Swallow, K. M., Zacks, J. M. & Abrams, R. A. Event Boundaries in Perception Affect Memory Encoding and Updating. *J. experimental psychology. Gen.* **138**, 236 (2009).

[19] Ben-Yakov, A. & Henson, R. N. The hippocampal film editor: sensitivity and specificity to event boundaries in continuous experience. *J. Neurosci.* **38**, 10057–10068 (2018).

[20] Reagh, Z. M., Delarazan, A. I., Garber, A. & Ranganath, C. Aging alters neural activity at event boundaries in the hippocampus and Posterior Medial network. *Nat. Commun.* **11**, 3980 (2020).

[21] Tulving, E. What is episodic memory? *Curr. Dir. Psychol. Sci.* **2**, 67–70 (1993).

[22] Scoville, W. B. & Milner, B. Loss of recent memory after bilateral hippocampal lesions. *J. Neurol. Neurosurg. & Psychiatry* **20**, 11–21 (1957).

[23] Burgess, N., Maguire, E. A. & O'Keefe, J. The human hippocampus and spatial and episodic memory. *Neuron* **35**, 625–641 (2002).

[24] Eichenbaum, H., Otto, T. & Cohen, N. J. The hippocampus—what does it do? *Behav. Neural Biol.* **57**, 2–36 (1992).

[25] McClelland, J. L., McNaughton, B. L. & O'Reilly, R. C. Why there are complementary learning systems in the hippocampus and neocortex: Insights from the successes and failures of connessionist models of learning and memory. *Psychol. rewiew* **102**, 419–457 (1995).

[26] Wheeler, M. E., Petersen, S. E. & Buckner, R. L. Memory's echo: Vivid remembering reactivates sensory-specific cortex. *Proc. Natl. Acad. Sci.* **97**, 11125–11129 (2000).

[27] Brodt, S. *et al.* Fast track to the neocortex: A memory engram in the posterior parietal cortex. *Science* **362**, 1045–1048 (2018).

[28] Michelmann, S., Bowman, H. & Hanslmayr, S. The temporal signature of memories: identification of a general mechanism for dynamic memory replay in humans. *PLoS Biol.* **14** (2016).

[29] Baldassano, C., Hasson, U. & Norman, K. A. Representation of real-world event schemas during narrative perception. *J. Neurosci.* **38**, 9689–9699 (2018).

[30] Hutchinson, J. B. & Barrett, L. F. The power of predictions: an emerging paradigm for psychological research. *Curr. Dir. Psychol. Sci.* (2019).

[31] Kuperberg, G. R. & Jaeger, T. F. What do we mean by prediction in language comprehension? *Lang. Cogn. Neurosci.* **31**, 32–59 (2016).

[32] Bar, M. The proactive brain: using analogies and associations to generate predictions. *Trends Cogn. Sci.* **11**, 280–289 (2007).

[33] Bar, M. The proactive brain: memory for predictions. *Philos. Transactions Royal Soc. B: Biol. Sci.* **364**, 1235–1243 (2009).

[34] Trapp, S. & Bar, M. Prediction, context, and competition in visual recognition: Predictions and competition. *Annals New York Acad. Sci.* **1339**, 190–198 (2015).

[35] Hindy, N. C., Ng, F. Y. & Turk-Browne, N. B. Linking pattern completion in the hippocampus to predictive coding in visual cortex. *Nat. Neurosci.* **19**, 665–667 (2016).

[36] Jafarpour, A., Piai, V., Lin, J. J. & Knight, R. T. Human hippocampal pre-activation predicts behavior. *Sci. Reports* **7**, 5959 (2017).

[37] Chen, J. *et al.* Accessing Real-Life Episodic Information from Minutes versus Hours Earlier Modulates Hippocampal and High-Order Cortical Dynamics. *Cereb. Cortex* **26**, 3428–3441 (2016).

[38] Lu, Q., Hasson, U. & Norman, K. A. Learning to use episodic memory for event prediction. preprint, Neuroscience (2020).

[39] Honey, C. J. *et al.* Slow cortical dynamics and the accumulation of information over long timescales. *Neuron* **76**, 423–434 (2012).

[40] Kösem, A. & Wassenhove, V. v. Distinct contributions of low- and high-frequency neural oscillations to speech comprehension. *Lang. Cogn. Neurosci.* **32**, 536–544 (2017).

[41] Pasley, B. N. *et al.* Reconstructing speech from human auditory cortex. *PLoS Biol.* **10**, e1001251 (2012).

[42] Lachaux, J.-P., Axmacher, N., Mormann, F., Halgren, E. & Crone, N. E. High-frequency neural activity and human cognition: Past, present and possible future of intracranial EEG research. *Prog. Neurobiol.* **98**, 279–301 (2012).

[43] Ray, S., Crone, N. E., Niebur, E., Franaszczuk, P. J. & Hsiao, S. S. Neural correlates of high-gamma oscillations (60-200 Hz) in macaque local field potentials and their potential implications in electrocorticography. *The J. Neurosci. The Off. J. Soc. for Neurosci.* **28**, 11526–11536 (2008).

[44] Di Liberto, G. M. *et al.* Cortical encoding of melodic expectations in human temporal cortex. *eLife* **9**, e51784 (2020).

[45] Holdgraf, C. R. *et al.* Rapid tuning shifts in human auditory cortex enhance speech intelligibility. *Nat. Commun.* **7**, 1–15 (2016).

[46] Tian, X., Ding, N., Teng, X., Bai, F. & Poeppel, D. Imagined speech influences perceived loudness of sound. *Nat. Hum. Behav.* **2**, 225–234 (2018).

[47] Broderick, M. P., Anderson, A. J. & Lalor, E. C. Semantic Context Enhances the Early Auditory Encoding of Natural Speech. *J. Neurosci.* **39**, 7564–7575 (2019).

[48] Goldstein, A. *et al.* Thinking ahead: prediction in context as a keystone of language in humans and machines. *bioRxiv* 2020.12.02.403477 (2020).

[49] Buhrmester, M., Kwang, T. & Gosling, S. D. Amazon's Mechanical Turk: A New Source of Inexpensive, Yet High-Quality, Data? *Perspectives on Psychol. Sci.* (2011).

[50] Zacks, J. M., Kumar, S., Abrams, R. A. & Mehta, R. Using movement and intentions to understand human activity. *Cognition* **112**, 201–216 (2009).

[51] Granger, C. W. J. Investigating causal relations by econometric models and cross-spectral methods. *Econometrica* **37**, 424–438 (1969).

[52] Barnett, L. & Seth, A. K. The MVGC multivariate granger causality toolbox: a new approach to granger-causal inference. *J. Neurosci. Methods* **223**, 50–68 (2014).

[53] Seth, A. K., Barrett, A. B. & Barnett, L. Granger causality analysis in neuroscience and neuroimaging. *J. Neurosci.* **35**, 3293–3297 (2015).

[54] Kass, R. E. & Raftery, A. E. Bayes Factors. *J. Am. Stat. Assoc.* **90**, 773–795 (1995).

[55] Ben-Yakov, A., Eshel, N. & Dudai, Y. Hippocampal immediate poststimulus activity in the encoding of consecutive naturalistic episodes. *J. Exp. Psychol. Gen.* **142**, 1255–1263 (2013).

[56] Stam, C. J., Nolte, G. & Daffertshofer, A. Phase lag index: Assessment of functional connectivity from multi channel EEG and MEG with diminished bias from common sources. *Hum. Brain Mapp.* **28**, 1178–1193 (2007).

[57] Mima, T., Matsuoka, T. & Hallett, M. Functional coupling of human right and left cortical motor areas demonstrated with partial coherence analysis. *Neurosci. Lett.* **287**, 93–96 (2000).

[58] Yeo, B. T. T. *et al.* The organization of the human cerebral cortex estimated by intrinsic functional connectivity. *J. Neurophysiol.* **106**, 1125–1165 (2011).

[59] Benjamini, Y. & Hochberg, Y. Controlling the false discovery rate: a practical and powerful approach to multiple testing. *J. Royal Statisitical Soc. Ser. B* **57**, 289–300 (1995).

[60] Genovese, C. R., Lazar, N. A. & Nichols, T. Thresholding of Statistical Maps in Functional Neuroimaging Using the False Discovery Rate. *NeuroImage* **15**, 870–878 (2002).

[61] Davachi, L. & DuBrow, S. How the hippocampus preserves order: the role of prediction and context. *Trends Cogn. Sci.* **19**, 92–99 (2015). ArXiv: 1011.1669v3 ISBN: 1364-6613.

[62] Staresina, B. P., Cooper, E. & Henson, R. N. Reversible Information Flow across the Medial Temporal Lobe: The Hippocampus Links Cortical Modules during Memory Retrieval. *J. Neurosci.* **33**, 14184–14192 (2013). Publisher: Society for Neuroscience Section: Articles.

[63] Clewett, D., DuBrow, S. & Davachi, L. Transcending time in the brain: How event memories are constructed from experience. *Hippocampus* **29**, 162–183 (2019).

[64] Zhang, H. *et al.* Gamma power reductions accompany stimulus-specific representations of dynamic events. *Curr. Biol.* **25**, 635–640 (2015).

[65] Lohnas, L. J. *et al.* Time-resolved neural reinstatement and pattern separation during memory decisions in human hippocampus. *Proc. Natl. Acad. Sci.* **115**, E7418–E7427 (2018).

[66] Teyler, T. J. & DiScenna, P. The hippocampal memory indexing theory. *Behav. Neurosci.* **100**, 147–154 (1986).

[67] Zheng, J. *et al.* Cognitive boundary signals in the human medial temporal lobe shape episodic memory representation. preprint, Neuroscience (2021).

[68] Fell, J. *et al.* Human memory formation is accompanied by rhinal–hippocampal coupling and decoupling. *Nat. Neurosci.* **4**, 1259–1264 (2001).

[69] Sols, I., DuBrow, S., Davachi, L. & Fuentemilla, L. Event boundaries trigger rapid memory reinstatement of the prior events to promote their representation in long-term memory. *Curr. Biol.* **27**, 3499–3504.e4 (2017).

[70] Kunz, L. *et al.* Hippocampal theta phases organize the reactivation of large-scale electrophysiological representations during goal-directed navigation. *Sci. Adv.* **5**, eaav8192 (2019).

[71] Lee, C. S., Aly, M. & Baldassano, C. Anticipation of temporally structured events in the brain. *bioRxiv* 2020.10.14.338145 (2020). Publisher: Cold Spring Harbor Laboratory Section: New Results.

[72] Michelmann, S., Staresina, B. P., Bowman, H. & Hanslmayr, S. Speed of time-compressed forward replay flexibly changes in human episodic memory. *Nat. Hum. Behav.* **3**, 143 (2019).

[73] Yuan, J. & Liberman, M. Speaker identification on the SCOTUS corpus. In *In Proceedings of Acoustics 2008* (2008).

[74] Yang, A. I. *et al.* Localization of dense intracranial electrode arrays using magnetic resonance imaging. *NeuroImage* **63**, 157–165 (2012).

[75] Rorden, C. & Brett, M. Stereotaxic display of brain lesions. *Behav. Neurol.* **12**, 191–200 (2000).

[76] Oostenveld, R., Fries, P., Maris, E. & Schoffelen, J.-M. FieldTrip: Open source software for advanced analysis of MEG, EEG, and invasive electrophysiological data. *Comput. Intell. Neurosci.* **2011**, 1–9 (2011).

[77] Michelmann, S. *et al.* Data-driven re-referencing of intracranial EEG based on independent component analysis (ICA). *J. Neurosci. Methods* **307**, 125–137 (2018).

[78] Fritsch, F. N. & Carlson, R. E. Monotone piecewise cubic interpolation. *SIAM J. on Numer. Analysis* **17**, 238–246 (1980).

[79] Ince, R. A. A. *et al.* A statistical framework for neuroimaging data analysis based on mutual information estimated via a gaussian copula. *Hum. Brain Mapp.* **38**, 1541–1573 (2017).

[80] Prichard, D. & Theiler, J. Generating surrogate data for time series with several simultaneously measured variables. *Phys. Rev. Lett.* **73**, 951–954 (1994).

[81] Pennington, J., Socher, R. & Manning, C. Glove: Global vectors for word representation. In *Proceedings of the 2014 Conference on Empirical Methods in Natural Language Processing (EMNLP)*, 1532–1543 (Association for Computational Linguistics, Doha, Qatar, 2014).

[82] Zwaan, R. A., Magliano, J. P. & Graesser, A. C. Dimensions of situation model construction in narrative comprehension. *J. Exp. Psychol. Learn. Mem. Cogn.* **21**, 386–397 (1995).

[83] Chung, S., Li, X. & Nelson, S. B. Short-Term Depression at Thalamocortical Synapses Contributes to Rapid Adaptation of Cortical Sensory Responses In Vivo. *Neuron* **34**, 437–446 (2002).
