# Supplementary material for: Moment-by-moment tracking of naturalistic learning and its underlying hippocampo-cortical interactions
Source: Nat Commun. 2021 Sep 13;12:5394. doi: 10.1038/s41467-021-25376-y (PMC8438040; doi:10.1038/s41467-021-25376-y)
Supplement: Supplementary file 3 — Reporting Summary [file 41467_2021_25376_MOESM3_ESM.pdf]

nature portfolio

Corresponding author(s): Sebastian Michelmann

Last updated by author(s): Jul 14, 2021

# Reporting Summary

Nature Portfolio wishes to improve the reproducibility of the work that we publish. This form provides structure for consistency and transparency in reporting. For further information on Nature Portfolio policies, see our Editorial Policies and the Editorial Policy Checklist.

## Statistics

For all statistical analyses, confirm that the following items are present in the figure legend, table legend, main text, or Methods section.

| n/a | Confirmed | |
|---|---|---|
| ☐ | ☒ | The exact sample size (*n*) for each experimental group/condition, given as a discrete number and unit of measurement |
| ☐ | ☒ | A statement on whether measurements were taken from distinct samples or whether the same sample was measured repeatedly |
| ☐ | ☒ | The statistical test(s) used AND whether they are one- or two-sided<br>*Only common tests should be described solely by name; describe more complex techniques in the Methods section.* |
| ☐ | ☒ | A description of all covariates tested |
| ☐ | ☒ | A description of any assumptions or corrections, such as tests of normality and adjustment for multiple comparisons |
| ☐ | ☒ | A full description of the statistical parameters including central tendency (e.g. means) or other basic estimates (e.g. regression coefficient) AND variation (e.g. standard deviation) or associated estimates of uncertainty (e.g. confidence intervals) |
| ☐ | ☒ | For null hypothesis testing, the test statistic (e.g. *F*, *t*, *r*) with confidence intervals, effect sizes, degrees of freedom and *P* value noted<br>*Give P values as exact values whenever suitable.* |
| ☒ | ☐ | For Bayesian analysis, information on the choice of priors and Markov chain Monte Carlo settings |
| ☒ | ☐ | For hierarchical and complex designs, identification of the appropriate level for tests and full reporting of outcomes |
| ☐ | ☒ | Estimates of effect sizes (e.g. Cohen's *d*, Pearson's *r*), indicating how they were calculated |

*Our web collection on statistics for biologists contains articles on many of the points above.*

## Software and code

Policy information about availability of computer code

| | |
|---|---|
| Data collection | Behavioral data were collected on millisecond.com using Inquisit 5 software. |
| Data analysis | Speech in the audio was time-stamped using Penn Phonetics Labs Foreced Aligner (P2FA, version 1.002, downloaded from: web.sas.upenn.edu). Data were analyzed in Matlab 2019b, using fieldtrip toolbox (development version, hash of github commit: 56769ab0c), gcmi : Gaussian-Copula Mutual Information (github.com/robince/gcmi) version 0.4, the MVGC Multivariate Granger Causality Toolbox (version: mvgc_v1.0, downloaded from sussex.ac.uk) and custom written code (https://github.com/s-michelmann/moment-by-moment-tracking) |

For manuscripts utilizing custom algorithms or software that are central to the research but not yet described in published literature, software must be made available to editors and reviewers. We strongly encourage code deposition in a community repository (e.g. GitHub). See the Nature Portfolio guidelines for submitting code & software for further information.

## Data

Policy information about availability of data

All manuscripts must include a data availability statement. This statement should provide the following information, where applicable:

- Accession codes, unique identifiers, or web links for publicly available datasets
- A description of any restrictions on data availability
- For clinical datasets or third party data, please ensure that the statement adheres to our policy

Behavioral data that underly the analyses in this manuscript and summary data from patients are available on Zenodo (doi: 10.5281/zenodo.5071942). Patient data in summarized form can reproduce the key figures and statistics in the manuscript. Because of their confidential nature, other patient data cannot be released to the public, but can be made available by the authors in de-identified form, upon reasonable request. Source data are provided with this paper as an Excel Sheet for

Figure 1b, Supplementary Figure 1c, Supplementary Figure 3b, and Supplementary Figure 13. Other figures are included in, or can be reproduced from the online data repository on Zenodo (doi: 10.5281/zenodo.5071942).

# Field-specific reporting

Please select the one below that is the best fit for your research. If you are not sure, read the appropriate sections before making your selection.

- [x] Life sciences
- [ ] Behavioural & social sciences
- [ ] Ecological, evolutionary & environmental sciences

For a reference copy of the document with all sections, see nature.com/documents/nr-reporting-summary-flat.pdf

# Life sciences study design

All studies must disclose on these points even when the disclosure is negative.

| | |
|---|---|
| Sample size | No statistical methods were used to determine the sample size. The number of patients was determined based on a trade-off between availability in the clinical setting, based on previous studies (e.g., Lohnas et al., PNAS, 2018; Honey et al. , Neuron, 2012) and constraints of feasibility. The number of behavioral participants was determined, based on previous experience with the behavioral prediction paradigm (Goldstien et al., 2020, BioRxiv). |
| Data exclusions | In order to improve the accuracy of group averages in the behavioral data, some subjects were excluded, if their responses deviated from the remaining group to an extent that exceeded a predefined threshold |
| Replication | The behavioral prediction experiment was replicated by collecting a new sample of the same size. The replication was successful, in that the new data expressed the same significant correlation with the neural data. |
| Randomization | In the behavioral prediction experiments, participants were randomly assigned to the two groups. For the event boundary norming task and the passive listening task performed by the patients, randomization is not relevant since there were no experimental groups. |
| Blinding | Blinding was not relevant in this study, because participants and experimenter do not directly interact in online-experiments. Patients were not assigned to conditions. |

# Reporting for specific materials, systems and methods

We require information from authors about some types of materials, experimental systems and methods used in many studies. Here, indicate whether each material, system or method listed is relevant to your study. If you are not sure if a list item applies to your research, read the appropriate section before selecting a response.

## Materials & experimental systems

| n/a | Involved in the study | |
|---|---|---|
| x | | Antibodies |
| x | | Eukaryotic cell lines |
| x | | Palaeontology and archaeology |
| x | | Animals and other organisms |
| | x | Human research participants |
| x | | Clinical data |
| x | | Dual use research of concern |

## Methods

| n/a | Involved in the study | |
|---|---|---|
| x | | ChIP-seq |
| x | | Flow cytometry |
| x | | MRI-based neuroimaging |

## Human research participants

Policy information about studies involving human research participants

| | |
|---|---|
| Population characteristics | Since the manuscript makes no conclusions regarding differences between participants, or correlations across participants, there are no covariate-relevant population characteristics in this study. Patients were 18-58 years old (mean = 28.78, SD = 11.82). 4 Patients were female, 5 right handed, 1 patient had unknown handedness. |
| Recruitment | Behavioral participants were recruited via Amazon Mechanical Turk. Patients were recruited via the Comprehensive Epilepsy Center of the New York University School of Medicine. Behavioral participants self-select to work on Amazon's mechanical Turk; their population has been described, for instance, by Walters et al., 2018, PLOS One. Patients self select by volunteering to participate in research experiments. |
| Ethics oversight | Ethical approval for the studies was granted by Princeton University Institutional Review Board, the Institutional Review Board at the New York University Langone Medical Center additionally approved the patient studies. Behavioral Participants provided informed consent before participation in accordance with the Princeton University Institutional Review Board. Patients provided informed consent in oral and written form before participation, in accordance with the Institutional Review Board at the New York University Langone Medical Center. |

Note that full information on the approval of the study protocol must also be provided in the manuscript.
